# Compressive Properties of Al-Si Alloy Lattice Structures with Three Different Unit Cells Fabricated via Laser Powder Bed Fusion

**DOI:** 10.3390/ma13132902

**Published:** 2020-06-28

**Authors:** Xiaoyang Liu, Keito Sekizawa, Asuka Suzuki, Naoki Takata, Makoto Kobashi, Tetsuya Yamada

**Affiliations:** 1 Department of Materials Process Engineering, Graduate School of Engineering, Nagoya University, Furo-cho, Chikusa-ku, Nagoya 464-8603, Japan; pigmentpig93@gmail.com (K.S.); suzuki.asuka@material.nagoya-u.ac.jp (A.S.); takata.naoki@material.nagoya-u.ac.jp (N.T.); kobashi.makoto@material.nagoya-u.ac.jp (M.K.); 2 Department of Space Flight Systems, JAXA (Japan Aerospace Exploration Agency), ISAS (Institute of Space and Astronautical Science), Yoshinodai, Chuo-ku, Sagamihara 252-5210, Japan; yamada.tetsuya@jaxa.jp

**Keywords:** powder bed fusion, lattice structure, AlSi10Mg alloy, mechanical property, finite element method

## Abstract

In the present study, in order to elucidate geometrical features dominating deformation behaviors and their associated compressive properties of lattice structures, AlSi10Mg lattice structures with three different unit cells were fabricated by laser powder bed fusion. Compressive properties were examined by compression and indentation tests, micro X-ray computed tomography (CT), together with finite element analysis. The truncated octahedron- unit cell (TO) lattice structures exhibited highest stiffness and plateau stress among the studied lattice structures. The body centered cubic-unit cell (BCC) and TO lattice structures experienced the formation of shear bands with stress drops, while the hexagon-unit cell (Hexa) lattice structure behaved in a continuous deformation and flat plateau region. The Hexa lattice structure densified at a smaller strain than the BCC and TO lattice structures, due to high density of the struts in the compressive direction. Static and high-speed indentation tests revealed that the TO and Hexa exhibited slight strain rate dependence of the compressive strength, whereas the BCC lattice structure showed a large strain rate dependence. Among the lattice structures in the present study, the TO lattice exhibited the highest energy absorption capacity comparable to previously reported titanium alloy lattice structures.

## 1. Introduction

Cellular lattice structures, consisting of a periodic arrangement of strut-based unit cells, exhibit numerous high-performance features, including ultra-light weight, high specific stiffness, thermal insulation and good energy absorption capability. These structures are of interest for the application in biomedical, automobile and aerospace fields [1,2,3]. For instance, Vilardell et al. reported Ti-6Al-4V lattice structures designed for implant applications with stiffness and density close to the host bone [4]. Cuadrado et al. [5] and Ahmadi et al. [6] studied the mechanical properties of cellular structures with different unit cells used for biomedical implant. Farzinazar et al. suggested that cellular structures offer promising candidates for thermal metamaterials based on the research findings [7]. Al-Saedi et al. investigated the mechanical behaviors of functionally graded lattice structures under compression loads, and highlighted their good energy absorption capability and potential for protective applications [8]. Recently, the Japan Aerospace Exploration Agency (JAXA) have proceeded with the Smart Lander for Investigating Moon (SLIM) project [9]. In the SLIM project, the lattice structures will be used as impact energy absorbers for landing of spacecrafts [10].

In the last decades, additive manufacturing (AM) technology has emerged as a frontier in the metal manufacturing process, owing to the great geometrical freedom for manufacturing specific structure (such as foams, honeycombs and lattices) with achievable complexity [11,12]. Laser powder bed fusion (LPBF), as one of the AM processes applicable to manufacturing metal products, can fabricate metallic lattice structures conveniently and effectively [13]. Nevertheless, lattice structures produced by the AM processes are expected to maximize their mechanical properties, while minimizing their weight in order to satisfy consumer-level requirements. The features affecting mechanical performance, including unit cell type, bulk material property, manufacturing process parameters, loading and boundary conditions, have drawn considerable attentions [14]. Leary et al. experimentally investigated the mechanical properties of stretch-dominated and bending-dominated lattice structures. It was found that the lattice structures with a high Maxwell number and struts parallel to the loading direction show high energy absorption and stiffness [15]. Suzuki et al. studied the effects of heat treatments on the compressive behaviors of the lattice structure of AlSi10Mg alloy, suggesting that the heat-treated lattice structures exhibit lower flow stress, but better ductility compared to the as-fabricated one [16]. Tsopanos et al. revealed the elastic modulus and yield strength of selective laser melted microlattice structures change linearly with laser power and laser exposure time. The linear correlations demonstrated that desired mechanical properties can be tailored via specific processing parameters [17]. The research findings by Choy et al. suggested that the different orientation of cubic and honeycomb cellular units also shows a significant effect on compressive and deformation behavior of lattice structures [18]. Several researches reported the investigation on characteristics of lattice structures under various applied loading conditions, such as drop weight [19], low-velocity impact [20], tensile loading [21], quasi-static and blast loading [22]. However, the effect of unit cell type on mechanical response of the lattice structures has been not sufficiently investigated for wide utilization as impact energy absorbers.

The present study aims to investigate the effect of unit cell type on compressive behavior of lattice structures. AlSi10Mg lattice structures with three different unit cells were fabricated by laser powder bed fusion. Mechanical properties and deformation behaviors were examined by compression experiment and FEM analysis. Additionally, the energy absorption properties of each lattice structure under practical conditions, assuming that spacecraft will land on the moon, were also studied.

## 2. Materials and Methods

### 2.1. Powder Characteristics and Fabrication Process

In the present study, an AlSi10Mg alloy (density: 2.67 g·cm^−3^) powder, fabricated by nitrogen gas atomization was selected for LPBF manufacturing, because the microstructure in bulk and lattice structures and bulk mechanical properties were clarified previously [23,24,25]. The microstructural inhomogeneity and anisotropy can be eliminated by a heat treatment. This alloy is appropriate for investigating the effect of unit cell type on the compressive properties of lattice structures. The compositions and morphology of the alloy powder are given in the previous report [23]. The lattice specimens used in this study were fabricated with EOSINT M 280 additive manufacturing system (EOS GmbH, Krailling, Germany), equipped with Yb-fiber laser, under an argon atmosphere and ambient temperature. The detailed processing parameters applied here were as follows: the laser power was 380 W, the bedded-powder layer thickness was 30 μm, the hatch distance was approximately 100 μm, and the rotation angle between layers was 67°. The schematic diagram of laser-scanning track used is given in the previous study [23]. The as-fabricated lattice specimen has a low ductility and hardly exhibits energy absorption properties [16]. To improve the ductility, the fabricated lattice specimens were annealed under a temperature of 300 °C, and for a period of 2 h, followed by slow furnace cooling.

### 2.2. Designs of Lattice Structure

**Figure 1:**
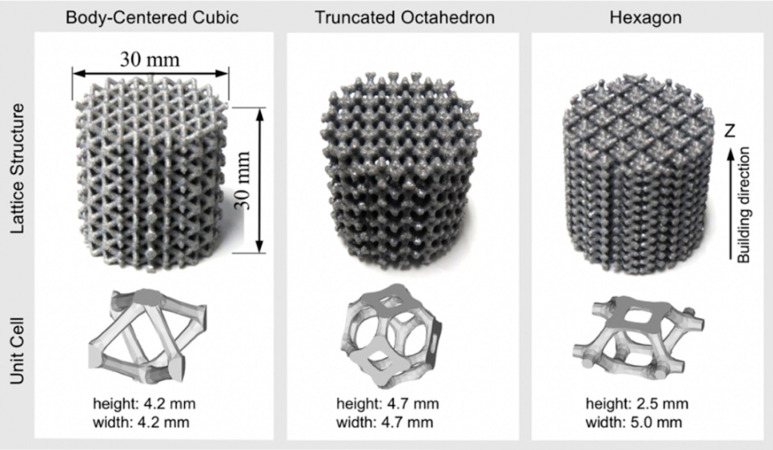
Photography images showing appearance of lattice structures and corresponding computer-aided design (CAD) models of body-centered cubic (BCC), truncated octahedron (TO), and hexagon (Hexa) unit cells.

Prior to manufacturing process, the computer-aided design (CAD) models with cylinder geometry were created by repeating unit cells. Figure 1 shows the appearance of fabricated lattice structures and the CAD models of the corresponding three different unit cells, including body-centered cubic (BCC), truncated octahedron (TO) and hexagon (Hexa) types. The diameter and height of specimens were 30 mm. Each type of lattice structures with different values of relative density (ρ*) were prepared. The relative densities (ρ*) of fabricated lattice specimens were measured by the Archimedes’ method. The measured relative densities (ρ*/*ρ*_s_) of all test specimens were as follows: 0.06, 0.16, 0.27 for the BCC type; 0.07, 0.17, 0.27 for the TO type; and 0.07, 0.18, 0.29 for the Hexa type.

### 2.3. Measurements

Quasi-static uniaxial compression tests were carried out on lattice specimens by a hydraulic universal testing machine (maximum load: 4.9 × 10^5^ N). According to ISO 13314 [26], compressive loading with an initial strain rate (2.2 × 10^−3^·s^−1^) was applied parallel to the building direction (Z direction), as shown in Figure 1. Nominal stress was obtained by the value of load divided by cross-section area, and nominal strain was obtained by the value of displacement divided by the height of lattice specimens. In order to capture the deformation behavior of compressed specimens, the compression test of the lattice structure specimens was interrupted at a compressive strain of approximately 20%, and then the deformed specimens were characterized by X-ray computed tomography (CT) using a TOSCANER-32252μhd (Toshiba, Tokyo, Japan) at a voltage of 180 kV and a current of 45 μA.

**Figure 2:**
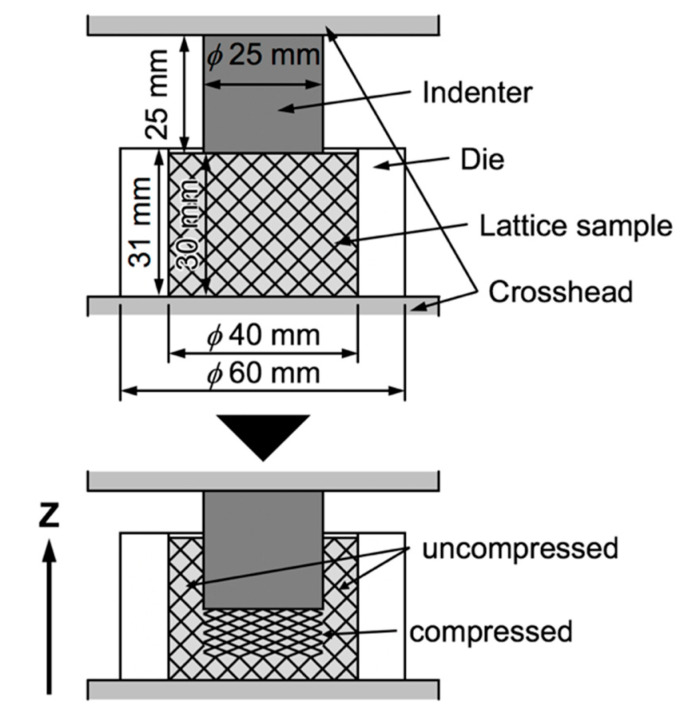
Schematic illustrations showing static indentation tests for lattice specimens.

**Figure 3:**
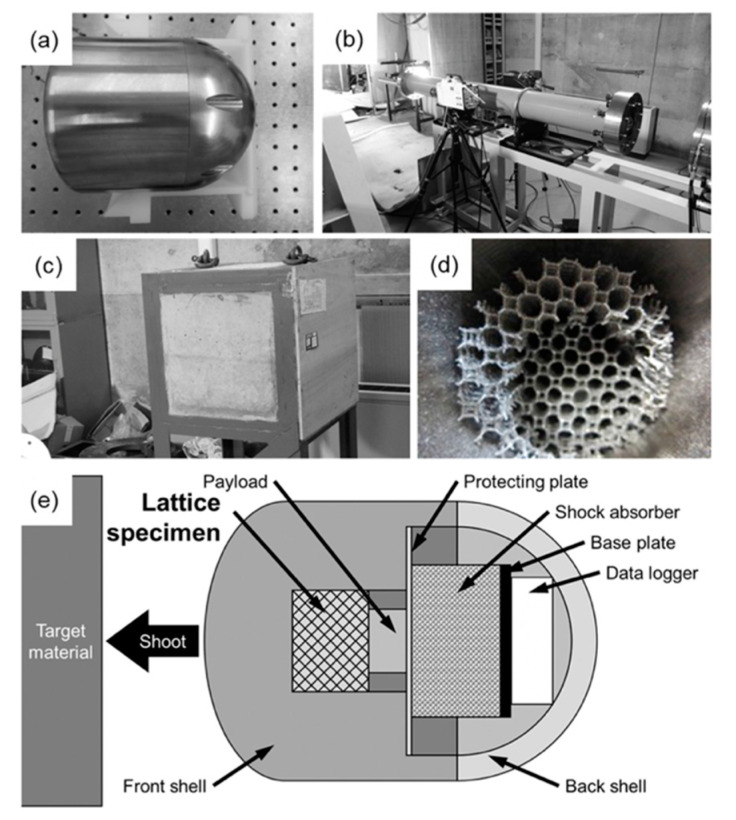
(**a**–**d**) Photography images showing the apparatuses and lattice specimen used for high-speed indentation tests: (**a**) crushable ball, (**b**) ballistic range, (**c**) target, and (**d**) TO lattice specimen pushed-in by payload. (**e**) Schematic illustration of the high-speed indentation tests.

When the lattice structures are applied as impact energy absorbers for spacecrafts, it is assumed that they are pushed-in by components of the spacecrafts at a high-speed. In order to obtain the mechanical properties of lattice structures in such a case, static and high-speed indentation tests were performed. Figure 2 presents the schematic illustrations showing the static indentation tests. The cylindrical lattice specimens with 30 mm in a height and 40 mm in a diameter set in a die (outer diameter: 60 mm, inner diameter: 40 mm, and height: 31 mm). The relative densities of the BCC, TO and Hexa lattice structures were 0.07. The indenter with 30 mm in a diameter was pushed into the lattice specimens in an initial strain rate of 2.2 × 10^−3^·s^−1^. The friction between the inner wall of the die and the lattice specimen was reduced by coating the inner wall with a lubricant, while the interface between the indenter and lattice specimen was not lubricated. Figure 3 shows (a–d) photography images showing apparatuses and lattice specimens used for the high-speed indentation tests and (e) schematic illustrations of the high-speed indentation tests. The cylindrical lattice specimens with 30 mm in a height and 40 mm in a diameter were set into the crushable balls (Figure 3a) and shot by the ballistic range (Figure 3b). The crushable ball was crashed to the target imitating the moon surface (Figure 3c) at a speed of approximately 27.0 m·s^−1^, which was approximately 4 × 10^5^ times faster than the static indentation tests (6.7 × 10^−5^ m·s^−1^). In the crushable ball, the payload was pushed into the lattice specimen (Figure 3d) by inertia and decelerated by the force received from the lattice specimen. The deceleration of the payload was measured with an accelerometer. Additionally, the deceleration of the payload was estimated from the static indentation properties, using the following equation of motion for the payload.

(1) $$m_{p}a_{p}=\sigma_{L}S_{p}\;,$$

where *m*_p_, *a*_p_, and *S*_p_ are the mass, deceleration, and bottom surface of the payload and *σ*_L_ is nominal stress loaded to the lattice specimen. The plateau stress obtained by the static indentation tests were substituted into Equation (1) to estimate the deceleration.

### 2.4. Finite Element Method

**Figure 4:**
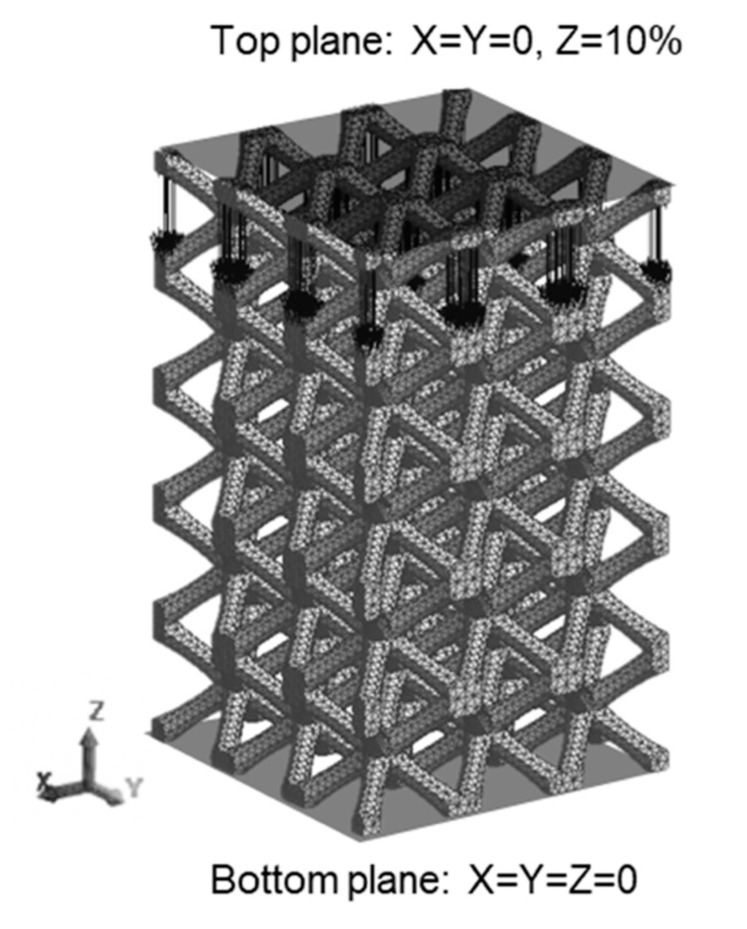
An example of FEM model (BCC lattice model) showing boundary conditions.

Elasto-plastic FEM analysis was carried out using a FEM software Femtet 2019. As shown in Figure 4, lattice models consisting of 3 × 3 × 5 unit cells were established, in view of the balance between the accuracy of the analysis and the computing time consumption. Tensile properties of the AlSi10Mg alloy, which is fabricated by the laser powder bed fusion and subsequently heat-treated at 300 °C for 2 h were used [23], and summarized in Table 1. A 4-node tetrahedron mesh with the element size of 0.3 mm was utilized. The top surfaces on each model were constrained in X and Y directions, bottom surfaces were fully constrained in all direction. Compressive displacements corresponding to 10% of heights of each model were applied on the top planes in the Z direction.

## 3. Results

### 3.1. Mechanical Behavior

**Figure 5:**
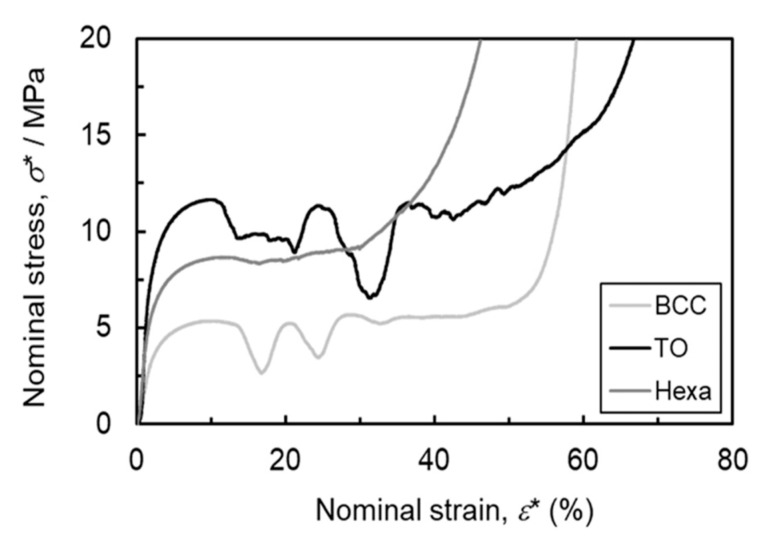
Nominal stress-strain curves of lattice structures consisting of the BCC (ρ*/*ρ*_s_ = 0.16), TO (ρ*/*ρ*_s_ = 0.17), and Hexa (ρ*/*ρ*_s_ = 0.18) unit cells.

**Figure 6:**
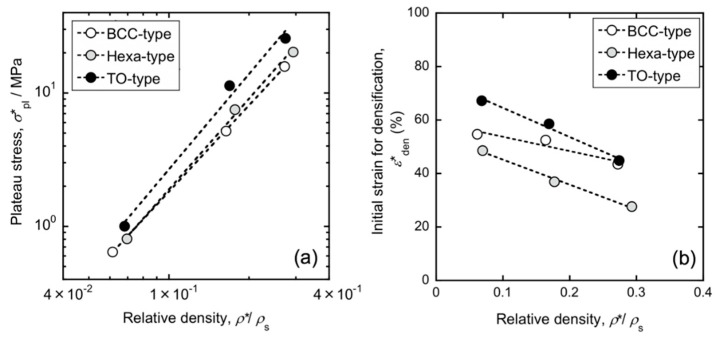
Changes in (**a**) plateau stress and (**b**) initial strain for densification as a function of relative density.

Figure 5 shows representative stress-strain curves of the lattice structures consisting of the BCC (ρ*/*ρ*_s_ = 0.16), TO (ρ*/*ρ*_s_ = 0.17), and Hexa (ρ*/*ρ*_s_ = 0.18) unit cells obtained by quasi-static uniaxial compression tests. The strain-stress curves of the lattice structures exhibited three distinct stages: elastic deformation with high linearity, plastic deformation and densification, as reported in several researches [4,27,28]. The TO lattice specimen exhibited the highest stiffness and yield strength, while the BCC lattice specimen did the lowest values. The strain-stress curves of BCC and TO lattice specimens exhibited stress fluctuation, whereas the flow curve of Hexa specimen show a steady plateau region. The Hexa lattice specimen was densified at a smaller strain (37%) than the BCC (53%) and TO (59%) lattice specimens. In accordance with ISO 13314 [26], the plateau stress is generally defined as an average value of the nominal stress in nominal strains ranging from 20% to 30%, and the initial strain for densification is defined as a nominal strain at which the flow stress rises to 1.3 times higher than the plateau stress. In this study, the measured average value of the nominal stress at a range of 5–10% in nominal strain, instead of 20–30%, was regarded as the plateau stress, because of the stress fluctuation observed at 20–30% in nominal strain for BCC and TO lattice specimens. Figure 6 shows changes in the (a) plateau stress and (b) initial strain for densification of all specimens as a function of the relative density. As relative density increases, the plateau stress increases almost linearly while the initial strain for densification decreases. The change in the plateau stress (*σ*_pl_*) as a function of the relative density (ρ*/*ρ*_s_) was regressed by the following Gibson-Ashby equation.

(2) $$\sigma_{pl}^{*}=C{\left(\frac{\rho^{*}}{\rho_{s}}\right)}^{n},$$

where *C* and *n* are constants. The values of *C* and *n* were summarized in Table 2. The power indexes (*n*) were in the range of 2.1~2.4. Kitazono reported that the power index of the TO lattice structure with a semi-spherical shape was approximately 2.1 [10]. The power indexes obtained in the present study almost coincided with the previous report. When comparing different types of specimens with similar relative densities, the TO lattice specimen (with higher plateau stress and initial strain for densification) rather than BCC and Hexa lattice specimens is favorable in terms of the energy absorption capacity.

### 3.2. Deformation Behavior

**Figure 7:**
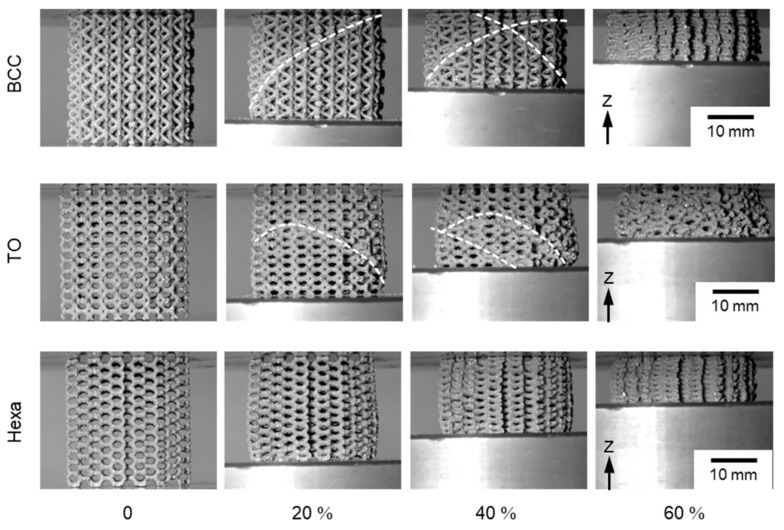
Photography images showing compression deformation behaviors of the lattice structures consisting of the BCC (ρ*/*ρ*_s_ = 0.16), TO (ρ*/*ρ*_s_ = 0.17), and Hexa (ρ*/*ρ*_s_ = 0.18) unit cells at 0%, 20%, 40%, and 60% nominal strains.

Figure 7 presents photography images showing the compressive deformation behaviors of the BCC (ρ*/*ρ*_s_ = 0.16), TO (ρ*/*ρ*_s_ = 0.17), and Hexa (ρ*/*ρ*_s_ = 0.18) lattice specimens during compression tests at different nominal strains of 0%, 20%, 40%, and 60%. The BCC and TO lattice specimens exhibited similar deformation behaviors and locally deformed in a direction of approximately 45° from the compressive axis. The local deformation corresponds to the formation of shear bands [8,16] leading to the first stress drop as shown in Figure 5. Another shear band occurred at the other local region in the further compressed specimens, resulting in the second stress drop. After that, the lattice structure specimens were continuously compressed into the densification. In the case of the Hexa lattice specimen, any shear band formations or localized fracture was not observed under the compression. An appearance of the compressed specimen showed a barrel-like shape, due to the friction between the lattice specimen and upper/lower plates for compression. Furthermore, the similar deformation behaviors were observed in each type of lattice specimens with various relative densities, suggesting that the deformation mechanism is strongly relevant to the unit cell type, rather than the relative density.

**Figure 8:**
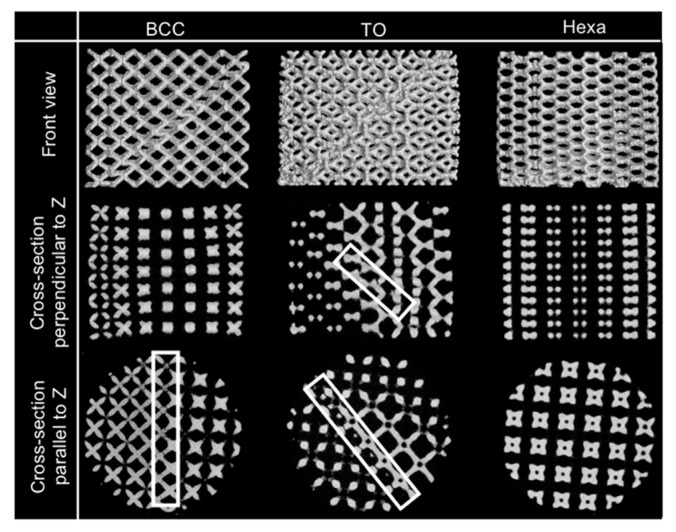
X-ray computed tomography (CT) reconstruction models and cross-section images of 20% compressed lattice structures.

**Figure 9:**
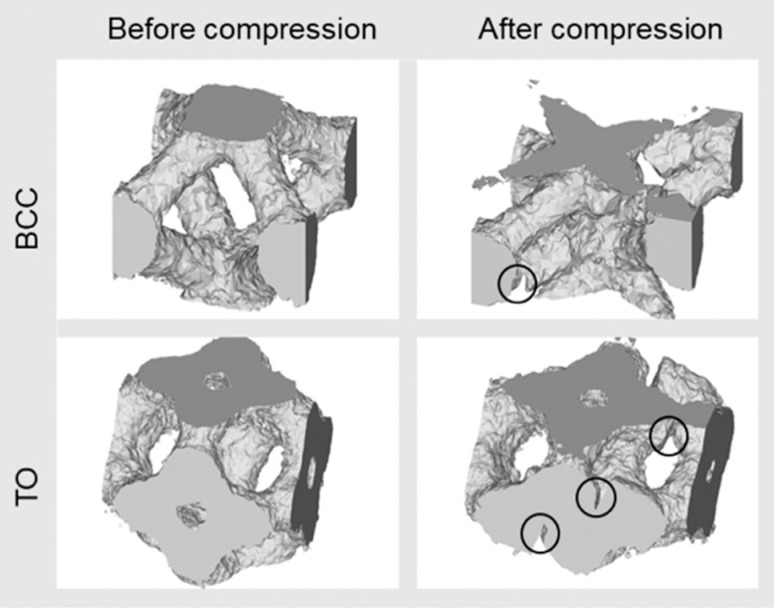
X-ray CT images of unit cells of BCC and TO lattice specimens, before and after compression.

The X-ray CT reconstruction models and the extracted cross-section images of 20% compressed lattice structures are shown in Figure 8. As shown in the front views of the three models, one shear band was formed in the BCC and TO lattice specimens at the strain, while the Hexa lattice specimen did not exhibit the shear band formation, which corresponded well to the compressive deformation behaviors shown in Figure 7. In the BCC and TO lattice specimens, which exhibited the shear band formations, cracks were generated in the struts on the same plane before compression, as shown by the square in the cross-sections. The crack initiation in the BCC lattice specimen was also reported in the previous study [16,29]. In the case of the Hexa lattice specimen without shear band formations, the cross-sectional geometries of the compressed Hexa lattice specimen (no shear band) appeared symmetric. No cracks were observed in the struts in a resolution level of the used X-ray CT. Thus, the observed initiated cracks were relevant to the shear band formations. To characterize the fractures of struts in BCC and TO lattice specimens, the inside unit cells were extracted from untested and tested X-ray CT models. The result is shown in Figure 9. It is clear that the cracks were initiated in the vicinity of nodes. The cracks in the vicinity of nodes in the BCC lattice structure were also observed in the previous study [16].

### 3.3. Static and High-Speed Indentation Tests

**Figure 10:**
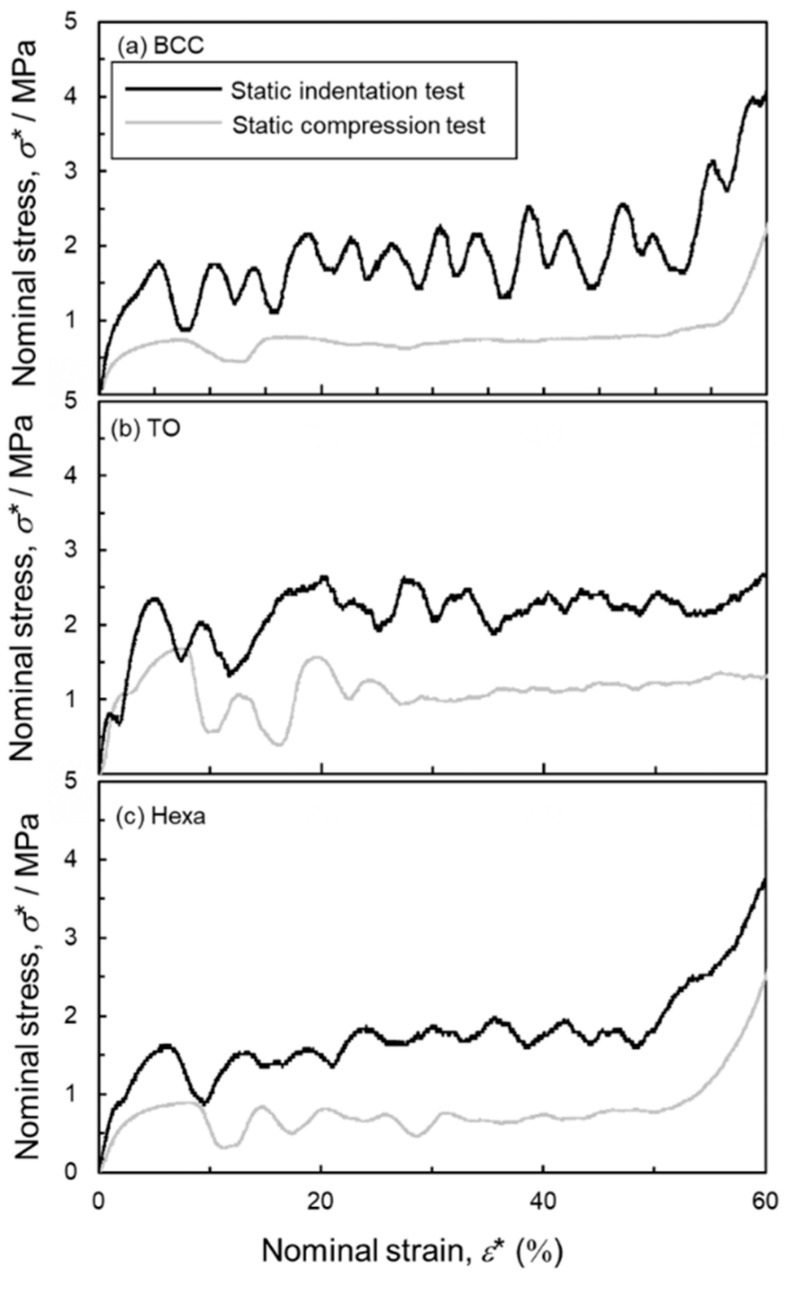
Nominal stress-strain curves measured by static compression and indentation tests of (**a**) BCC, (**b**) TO and (**c**) Hexa lattice specimens.

Figure 10 shows the nominal stress-strain curves of the (a) BCC, (b) TO and (c) Hexa lattice specimens evaluated by the static compression and indentation tests. The indentation strengths of each lattice structure were higher than the compressive strength. The flow stress in the plateau regions in the indentation stress-strain curves fluctuated more frequently than that in the compressive stress-strain curves. This is because the indenter needs to shear fracture the struts between the compressed and uncompressed regions. The stress fluctuations were caused by the shear band formation and its associated shear fracture of the struts. Except for the above, the indentation properties of each lattice structures exhibited similar trend to the compressive properties.

**Figure 11:**
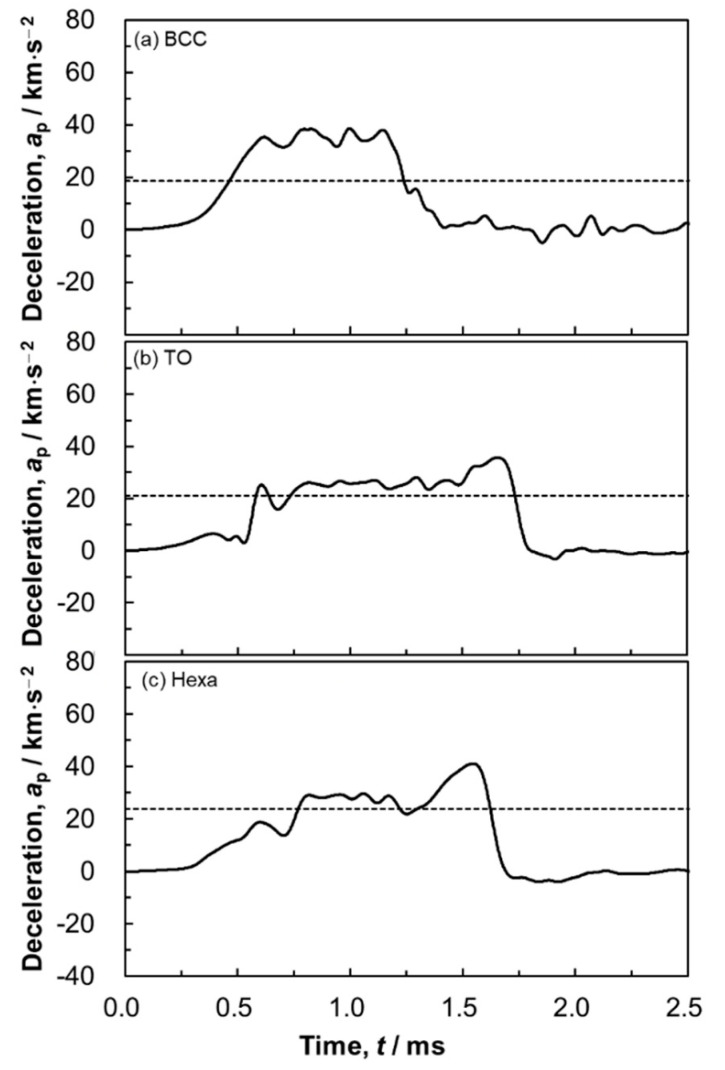
Change in acceleration measured by the high-speed indentation tests as a function of time: (**a**) BCC, (**b**) TO and (**c**) Hexa lattice specimens. The horizontal broken lines indicate the acceleration estimated from the plateau stress in the stress-strain curves of static indentation tests shown in Figure 10.

Figure 11 presents change in the deceleration during the high-speed indentation tests of the (a) BCC, (b) TO and (c) Hexa lattice specimens as a function of time. The deceleration increased with time and became roughly constant after approximately 0.5 ms. After certain periods, the decelerations decreased to 0 km·s^−2^. Based on Equation (1), the constant decelerations indicate that the lattice structures deformed under constant stresses (plateau region). The horizontal broken lines in the figures indicate the decelerations estimated from the plateau stresses of each lattice structures obtained by static indentation tests (Figure 10), using Equation (1). Although the experimental deceleration in the case of the BCC lattice specimen was higher than estimated value, the estimated decelerations in the case of TO and Hexa lattice specimens were in good agreement with the experimental values. These results indicate that the energy absorption properties of the TO and Hexa lattice specimens in practical conditions could be predicted based on the results of static tests. However, the reason for different strain rate dependence depending on the unit cell morphology remains unclear. Systematical compressive tests under various strain rates need to be carried out in the future.

## 4. Discussion

### 4.1. Compressive Behavior

**Figure 12:**
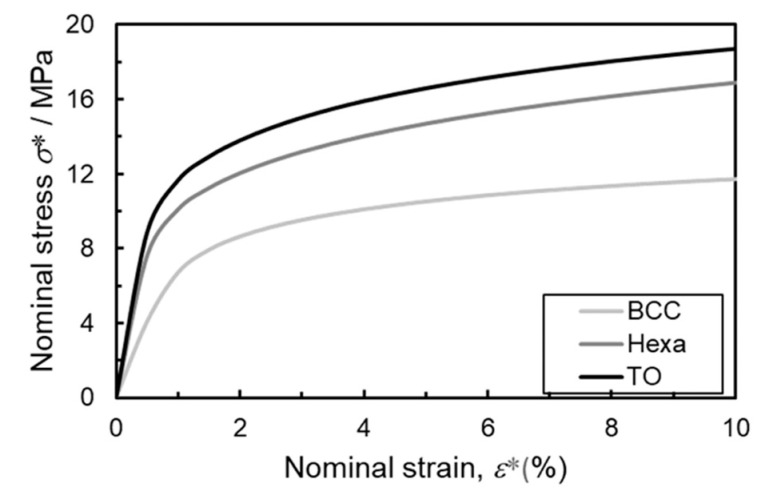
Stress-strain curves for lattice models consisting of the BCC, TO and Hexa unit cells calculated by the FEM analysis.

**Figure 13:**
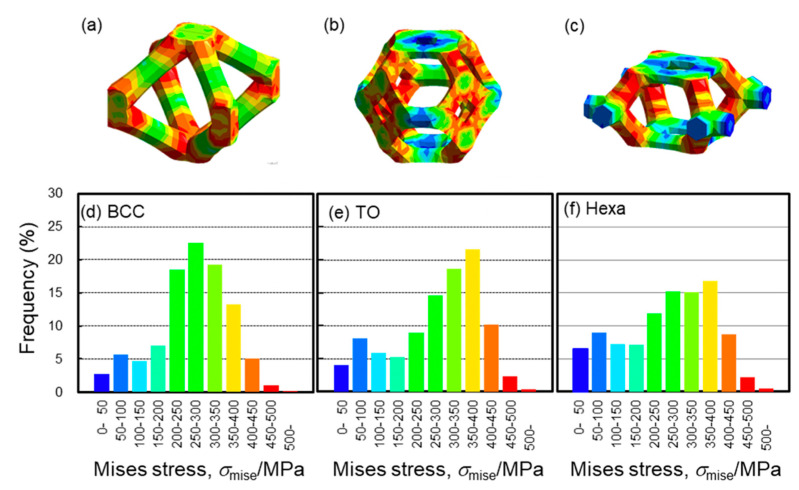
(**a**–**c**) Contour maps and (**d**–**f**) histograms showing the Mises stress distributions in BCC, TO and Hexa models obtained by of FEM analysis. The colors for bars in the histograms correspond to the those of the contour maps.

The plateau stress of the TO lattice specimen was the highest in the lattice structures in this study (Figure 5 and Figure 6a). In order to elucidate the reason for the higher plateau stress, the FEM analysis was conducted. Figure 12 presents the calculated stress-strain curves for the lattice models consisting of the BCC, TO, and Hexa unit cells simulated by the FEM analysis. The compressive strength of the TO lattice model was higher than the BCC and Hexa lattice models, which is in good agreement with the experimental results (Figure 5 and Figure 6a). Figure 13 presents (a–c) contour maps and (d–f) histograms showing the Mises stress distribution in each lattice structure at a nominal strain of 10%. The colors of the bars in the histograms correspond to those of the contour maps. In the TO and Hexa lattice models, the struts oriented perpendicular to Z direction showed low level of stress (Figure 13b,c). The stresses of the oriented struts (not perpendicular to Z direction) were high and relatively uniform (Figure 13b,c). The BCC lattice model does not have such struts perpendicular to Z direction, but has elongated struts with a high aspect ratio comparted with the TO and Hexa models. In the struts, highly stressed regions were often localized at the parts in the vicinity of nodes rather than the center parts (Figure 13a). Mises stresses of the BCC, TO and Hexa lattice models varied from 0 to 600 MPa. Mises stress in the range of 200–400 MPa dominated in the BCC model. The stress of 200–400 MPa corresponded the stress generated in the center parts of the struts shown in Figure 13a. Most elements in the TO model showed high Mises stress of 300–450 MPa. Additionally, in the case of the Hexa model, the fraction of the Mises stress in 300–450 MPa was high, but the Mises stress below 250 MPa was also frequently observed. This is because the Hexa lattice model has more struts oriented perpendicular to the Z direction than the BCC and TO lattice models. The results indicated that the TO specimen can bear high stress uniformly, resulting in high stiffness and plateau stress, as shown in Figure 5 and Figure 12. In order to enhance the compressive strength of the lattice structures, the design of unit cells consisting of many low aspect ratio struts not oriented perpendicularly to the compressive direction is required.

**Figure 14:**
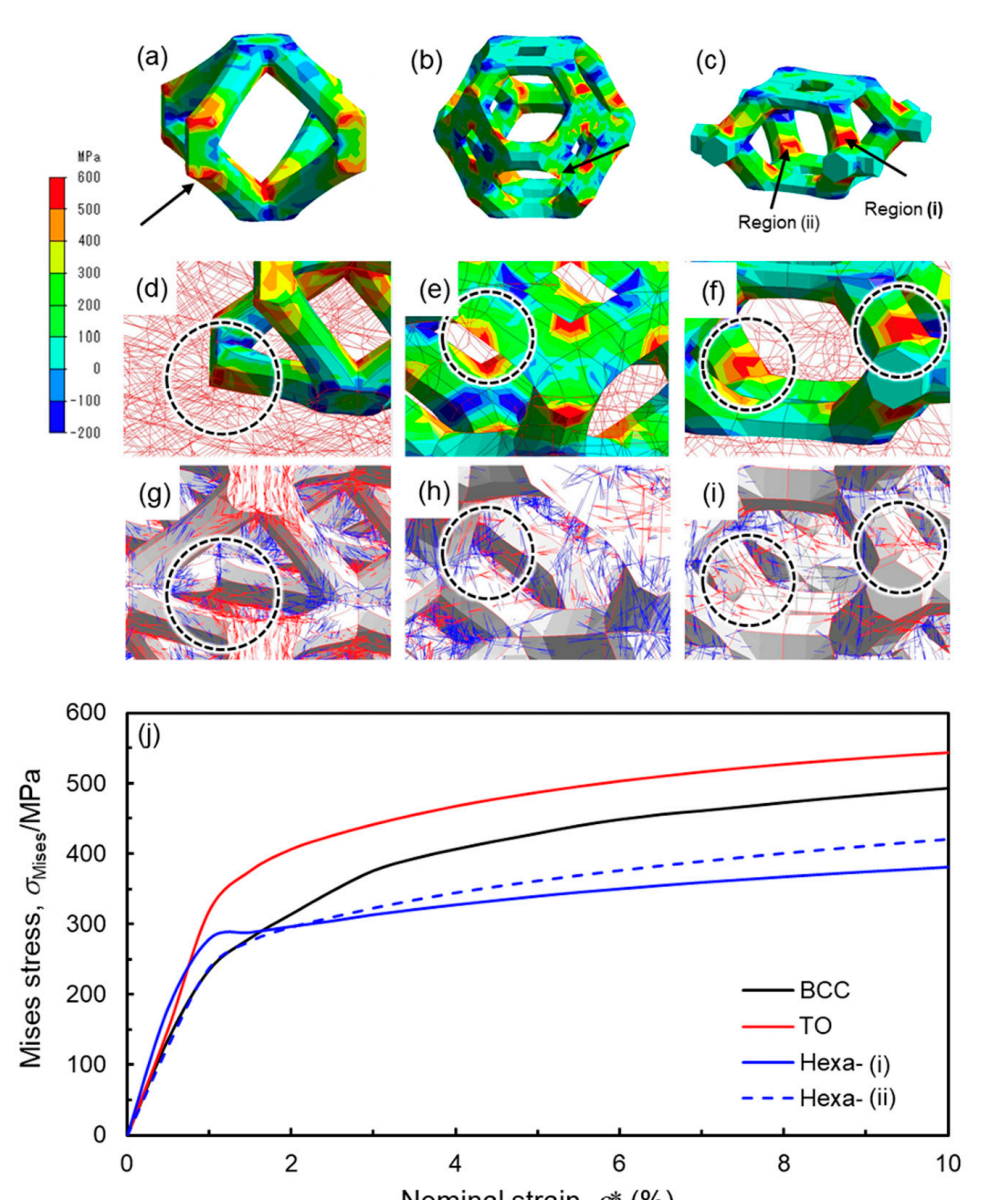
(**a**–**f**) Maximum principal stress distributions and (**g**–**i**) surface vectors of principal stresses in the (**a**,**d**,**g**) BCC, (**b**,**e**,**h**) TO and (**c**,**f**,**i**) Hexa lattice models. (**j**) Change in the Mises stress at the positions marked by arrows in (**a**–**c**).

The Hexa lattice specimens exhibit no stress drops caused by the shear band formation (Figure 5 and Figure 7). It is reported that the shear band formation in the BCC lattice structure induced bending deformation of struts, resulting in crack initiations on tensile stress parts [16,30]. In fact, the cracks were observed in X-ray CT images of the BCC and TO lattice specimens (Figure 8 and Figure 9). These results suggest that the shear band formation was relevant to crack initiations, due to local stress concentrations. Figure 14a–c shows the maximum principal stress distribution in the (a) BCC, (b) TO and (c) Hexa unit cells. In the BCC and TO lattice model, high maximum principal stresses with positive values arose at the arrow-marked positions (Figure 14a,b). The positions almost corresponded to the crack initiation positions shown in Figure 9. Additionally, Figure 14d–i presents the (d–f) magnified maximum principal stress distribution and (g–i) surface vectors of the principal stresses in the struts of the (d,g) BCC, (e,h) TO and (f,i) Hexa unit cells marked by the arrows. The maximum principal stress in the struts in the BCC and TO models had tensile directions along the struts (Figure 14d,e,g,h). It is considered that such high tensile stress generated cracks in the struts, resulting in the shear band deformation. In the case of the Hexa lattice model (Figure 14c), high maximum principal stress with positive values arose in the struts near nodes, as marked by the arrows (regions (i) and (ii)). At the positions, the maximum principal stress had tensile directions along struts (Figure 14f,i). Figure 14j shows the relationship between the Mises stress at the arrow-marked positions and nominal strain. The Mises stresses concentrated at regions (i) and (ii) in the Hexa model were lower than those of the crack initiation regions in BCC and TO models, which is in agreement with the less tendency of crack initiations in the Hexa lattice structure. It is considered that the moderate stress concentration suppressed the shear band formation, as shown in Figure 7. However, in order to essentially elucidate the reason why the Hexa lattice structures did not exhibit the shear band formation, it is required to identify the process of the shear band formation in the future.

**Figure 15:**
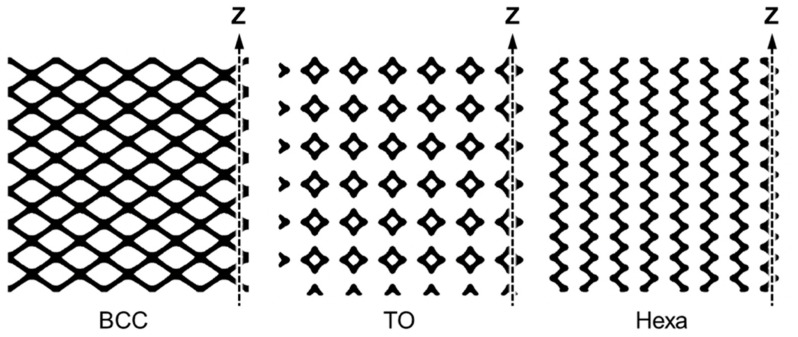
Cross-sections of CAD models showing the BCC, TO and Hexa lattice structures. These cross-sections include the most struts in each lattice structure.

The initial strain for densification of the Hexa lattice specimen was the smallest among the lattice structures in this study (Figure 5 and Figure 6b). The densification is initiated by overlapping adjacent struts in the loading direction. Figure 15 illustrates the CAD models showing the cross-sections of the three specimens. The cross-section includes the most struts in each lattice structure. The numbers of struts on the broken arrows in the BCC, TO and Hexa lattice specimens are 14, 13 and 22, respectively. Densifications are expected to start from contacts between struts. Therefore, the nominal strains for strut contacts (ε*_con_) in each lattice structure were geometrically estimated using the following equation.

(3) $$\varepsilon_{con}^{*}=1-\frac{t\times N}{h}\;,$$

where *t* is the diameter of the struts, *N* is the number of struts on the dotted arrows in Figure 15, and *h* is the height of the lattice specimens. The estimated the nominal strains for strut contacts were 59% for the BCC (ρ*/*ρ*_s_ = 0.16) lattice specimen, 66% for the TO (ρ*/*ρ*_s_ = 0.17) lattice specimen, and 34% for the Hexa (ρ*/*ρ*_s_ = 0.18) lattice specimen, which was in good agreement with experimental initial strains for densification (53% for the BCC, 59% for the TO, and 37% for the Hexa) shown in Figure 6b. Thus, the contact of struts in Hexa lattice specimen would happen earlier under compression, due to the larger amount of struts in Z direction, resulting in early densification.

### 4.2. Energy Absorption Capacity

**Figure 16:**
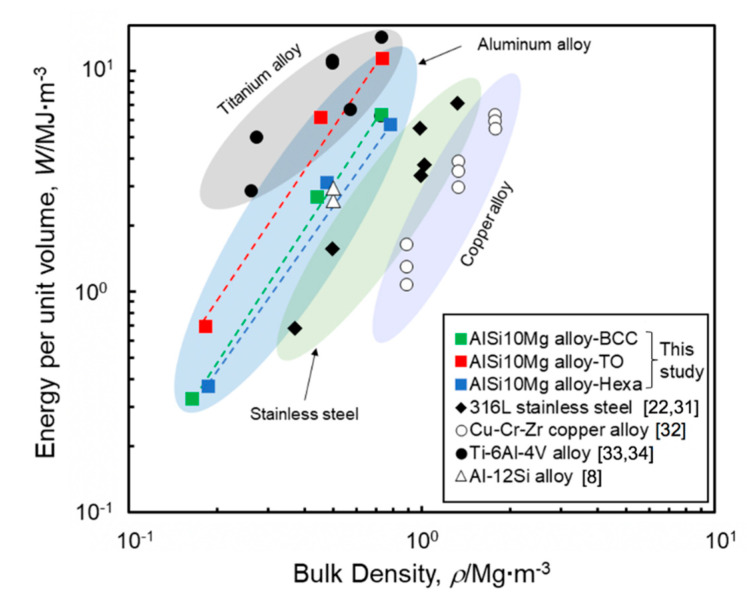
Relationship between energy per unit volume and bulk density of AlSi10Mg lattice structures. For comparison, the results of various lattice structures consisting of different materials are also shown in the Figure.

The energy absorption capacities of lattice structures in this study are discussed. Figure 16 shows changes in energy absorption capacities of lattice structures consisting of different materials as a function of the bulk density. The AlSi10Mg lattice structures with the BCC, TO, and Hexa unit cells were shown together with various lattice structures made of 316L stainless steel [22,31], Cu-Cr-Zr copper alloy [32], Ti-6Al-4V alloy [2,33] and Al-12Si alloy [8]. Aluminum alloy lattice structures exhibit a lower energy absorption capacity compared to titanium alloy lattice structures, but a higher capacity than stainless steel and copper alloy lattice structures. The AlSi10Mg lattice structures with the BCC and Hexa unit cells exhibited almost the same capacity of the Al-12Si lattice structure [8]. On the other hand, the maximum value of energy absorption capacity in this study is 11.4 MJ·m^−3^, achieved by the TO lattice structure with the relative density of 0.27, which significantly exceeds the energy absorption capacity of the BCC and Hexa lattice structures. The performance of AlSi10Mg TO lattice structure reaches the level of titanium alloy lattice structures, indicating that the AlSi10Mg lattice structures with the TO-type unit cell could serve as substitute for expensive titanium alloy lattice structures, without sacrificing the energy absorption capacity. The superior energy absorption capacity of the TO lattice structure (Figure 16) would be applied to the other materials, indicating the potential improvement of the performance of the titanium alloy lattice structure with the TO-type unit cell.

## 5. Conclusions

In this study, AlSi10Mg lattice structures with different unit-cell types consisting of body-centered cubic (BCC), truncated octahedron (TO) and hexagon (Hexa) geometry were fabricated via laser powder bed fusion (LPBF). The effects of unit cell type on mechanical properties and deformation behaviors of lattice structures were investigated and analyzed by compression tests, X-ray CT observation and FEM analysis. Accordingly, the main conclusions can be drawn as follows.

1. Experimental results reveal that TO lattice specimen exhibited higher stiffness and plateau stress in comparison to BCC and Hexa lattice specimen. Based on the stress distribution from FEM analysis, most elements in the TO model showed high Mises stress, while low Mises stress dominated in the BCC model.
2. During the compression test, two sequential diagonal shear bands were observed in the BCC and TO specimen, along with the fluctuation of abrupt stress drops. Cracks and fracture of struts in severely deformed unit cells were found in X-ray CT images. However, Hexa specimen experienced continuous deformation behavior, and inside struts remained intact. The low concentrated Mises stress in Hexa lattice model contributed to the deformation behavior without shear band formation.
3. The Hexa lattice specimen densified at a smaller strain. Multiple struts in loading direction in the Hexa lattice specimen resulted in struts overlapping and early densification.
4. High-speed indentation tests revealed that the energy absorption properties of the TO and Hexa lattice specimens under practical conditions could be predicted by static indentation tests.
5. Aluminum alloy lattice structures exhibited higher energy absorption capacity compared to the stainless steel and copper alloy lattice structures. The AlSi10Mg lattice structures with the TO unit cell showed their superiority in absorbing energy reaching the level of titanium alloy lattice structures.

## Figures and Tables

**Table 1:** Material properties used for FEM analysis. The properties were determined based on the tensile properties of the AlSi10Mg alloy, which is fabricated by the laser powder bed fusion and subsequently heat-treated at 300 °C for 2 h [23].

**Young’s modulus, E/GPa**	60
**Poisson’s ratio,** ν	0.3
**Yield stress, *σ_YS_*/MPa**	200
**Work hardening coefficient, n**	0.2

**Table 2:** Constants for the BCC, TO, and Hexa lattice specimens in the Gibson-Ashby equation.

	C/MPa	n
BCC	257	2.1
TO	345	2.3
Hexa	633	2.4
